# Immune Response Induced by an Immunodominant 60 kDa Glycoprotein of the Cell Wall of *Sporothrix schenckii* in Two Mice Strains with Experimental Sporotrichosis

**DOI:** 10.1155/2016/6525831

**Published:** 2016-03-14

**Authors:** Carlos A. Alba-Fierro, Armando Pérez-Torres, Conchita Toriello, Evelyn Pulido-Camarillo, Everardo López-Romero, Yolanda Romo-Lozano, Gerardo Gutiérrez-Sánchez, Estela Ruiz-Baca

**Affiliations:** ^1^Facultad de Ciencias Químicas, Universidad Juárez del Estado de Durango, Avenida Veterinaria S/N, 34120 Durango, DGO, Mexico; ^2^Departamento de Biología Celular y Tisular, Facultad de Medicina, Universidad Nacional Autónoma de México, 04510 Ciudad de México, DF, Mexico; ^3^Departamento de Microbiología y Parasitología, Facultad de Medicina, Universidad Nacional Autónoma de México, 04510 Ciudad de México, DF, Mexico; ^4^Departamento de Biología, División de Ciencias Naturales y Exactas, Universidad de Guanajuato, Campus Guanajuato, Noria Alta S/N, 36050 Guanajuato, GTO, Mexico; ^5^Departamento de Microbiología, Centro de Ciencias Básicas, Universidad Autónoma de Aguascalientes, Avenida Universidad No. 940, 20131 Aguascalientes, AGS, Mexico; ^6^Department of Biochemistry and Molecular Biology and Complex Carbohydrate Research Center, University of Georgia, 315 Riverbend Road, Athens, GA 30602-4712, USA

## Abstract

Cell wall (CW) components of fungus* Sporothrix schenckii* are the major inductors antigens of immune responses. The immunodominant 60 kDa glycoprotein (gp60) has been shown to be associated with the virulence of this fungus but its role in experimental sporotrichosis is unknown. In this work, the immunological effects of CW-purified gp60 were investigated in a model of experimental subcutaneous sporotrichosis in normal and gp60-preimmunized C57BL/6 and BALB/c mice strains which were then infected with* S. schenckii* conidia. Results showed that both mice strains use different cytokine profiles in order to fight* S. schenckii* infection; C57BL/6 mice seem to use a Th17 response while BALB/c mice tend to depend on a Th1 profile. Preimmunization with gp60 showed a downregulatory effect on the immune response since cytokines levels were diminished in both strains. There were no significant differences in the magnitude of dorsoplantar inflammation between gp60-preimmunized and nonimmunized mice of both strains. However, skin lesions due to the infection in gp60-preimmunized mice were more severe in BALB/c than in C57BL/6 mice, suggesting that the antigen exerts a higher downregulatory effect on the Th1 response.

## 1. Introduction

Sporotrichosis is a chronic mycosis that affects skin and subcutaneous tissues, but it also can spread to other organs through the lymph vessels [1–4]. It is acquired by traumatic implantation of the etiologic agent, the dimorphic fungus* Sporothrix schenckii*. The mycelial morphotype is found in soil, wood, and plants [5], while the yeast morphotype is found in host tissues [6, 7]. Surface components of* S. schenckii* cell wall (CW) have a possible role in its pathogenicity. Accordingly, ergosterol peroxide helps the organism to evade the host's immune response, promoting fungal infection [8]. Also, a lipid antigen has been shown to decrease the production of proinflammatory cytokines such as TNF-*α*, IL-1*β*, and also IL-12 and IL-10 in experimental models of sporotrichosis [9, 10]; moreover, it is capable of inhibiting macrophage phagocytosis in* in vitro* assays [11].

One of the most studied CW components of* S. schenckii* is a peptide-polysaccharide known as peptide-rhamnomannan, a glycoconjugate molecular complex with a wide range of molecular weights composed of 33.5% rhamnose, 57% mannose, and 14.2% protein [12]. In murine models of sporotrichosis, the peptide-rhamnomannan is involved in the anti-inflammatory response diminishing the production of IL-1*β* and TNF-*α* [10], and in* in vitro* lymphoproliferation assays have shown that it contains components with different mitogenic activities [13–15].

Recently, a 70 kDa glycoprotein (gp70) isolated from the CW of the yeast morphotype of* S. schenckii* has become a relevant cell surface component [16]. This molecule has an isoelectric point (IP) of 4.1 and about 5.7% of its molecular weight (MW) corresponds to carbohydrate residues. Some important features of gp70 are its ability to adhere to extracellular matrix proteins [16] and to induce a specific humoral response in* S. schenckii *infected mice [17]. Interestingly, administration of anti-gp70 monoclonal antibodies appears to have protective effect against fungal infection in mice [18]. In addition to the antigenic gp70,* S. schenckii* strains also express a 60 kDa immunodominant glycoprotein (gp60) [19, 20], and both have been proposed as potential virulence factors since they are expressed by the most virulent strains of the* Sporothrix* complex [21]. Recent studies seem to indicate that gp70 and gp60 share the same peptide but differ in glycosylation pattern, IP, and MW [22].

In order to shed light on the role of the gp60 during infection by* S. schenckii*, the antigen was purified by isoelectric focusing and continuous elution electrophoresis, and its effect on the immune response in two mice strains with experimental cutaneous sporotrichosis was evaluated.

## 2. Materials and Methods

### 2.1. Bioethical Statements

All procedures carried out in animals were approved by the Animal Ethics Committee, Facultad de Medicina, Universidad Nacional Autónoma de México, and followed the Mexican Official Guide (NOM 062-ZOO-1999) for the care and use of laboratory animals.

### 2.2. Animals

Male C57BL/6 and BALB/c mice, 8–10 weeks old, were purchased from Harlan (Mexico City, Mexico). The animals were free of parasites or pathogens and were fed mouse chow (Purina de México, México) and water* ad libitum*. Mice were housed in separate cages with wood shavings as nesting material, and five individuals were housed per cage. They were maintained in a 12/12 h light/dark cycle in a room thermostatically maintained at 24 ± 2°C throughout the study. Groups of ten experimental animals by mice strain and each corresponding control group of five mice were conformed. Two 12-week-old male New Zealand rabbits weighing 3.21 and 3.35 Kg were used to obtain hyperimmune serum.

### 2.3. Organism and Culture Conditions

*S. schenckii* strain ATCC 58251 was used for this study. Conidia were obtained from the mycelial morphotype prepared from a 10-day-old culture grown at 28°C in YPG medium [0.3% (w/v) yeast extract, 1% (w/v) peptone, and 2% (w/v) glucose] at pH 4.5. The yeast morphotype was obtained in YPG medium, pH 7.2, inoculated with 5 × 10^5^ conidia mL^−1^, and incubated for 10 days at 37°C with shaking (120 rpm). The harvested cells (centrifugation at 7000 g for 10 min) were washed twice with lysis buffer [50 mM Tris-HCl, pH 7.5, supplemented with 1 mM phenylmethylsulfonyl fluoride (PMSF)] and maintained at −20°C until used.

### 2.4. Extraction of CW Proteins

Yeast cells were resuspended in lysis buffer and broken with glass beads (0.45–0.5 mm in diameter) in an MSK cell homogenizer (Braun Melsungen, Germany) by alternate periods of breaking (40 s) and cooling (60 s) until all cells were broken. To isolate the CW, the cell homogenate was centrifuged at 1300 g for 15 min at 4°C, and the pellet was washed thrice with lysis buffer to remove any intracellular component associated with the CW during the cell-breaking process. CW proteins were extracted with hot 2% (w/v) sodium dodecyl sulfate (SDS) as described previously [16], precipitated with 70% ethanol for 2 h at −20°C, and stored at −70°C until use. CW proteins were quantified with the DC kit (Bio-Rad).

### 2.5. Two-Dimensional Gel Electrophoresis (2D-PAGE)

CW proteins were analyzed by 2D-PAGE gels as described by Ruiz-Baca et al. [19]. Briefly, the extracted proteins were cleaned with the Readyprep 2D Cleanup kit (Bio-Rad) following the manufacturer's instructions. Samples of 160 *μ*g of protein were resuspended in hydration buffer [7 M urea, 2 M thiourea, 4% (w/v) 3-[(3-cholamidopropyl)dimethylammonio]-1-propanesulfonate (CHAPS), 20 mM dithiothreitol (DTT), 0.5% (w/v) ampholytes, and 0.002% (w/v) bromophenol blue] and applied on immobilized pH 4–7 gradient strips (7 cm, Bio-Rad). The samples were hydrated for 16 h at 4°C. Isoelectric focusing (IEF) was performed in a Protean IEF system (Bio-Rad) using the following conditions: 250 volts (V) for 20 min, 4000 V for 2 h until 10000 V/h was reached. After IEF, the strips were incubated sequentially for 15 min in equilibrium buffer I [50 mM Tris/HCl, pH 8.8, 6 M urea, 30% (v/v) glycerol, 2% (w/v) SDS, and 0.5% (w/v) DTT] and equilibrium buffer II [50 mM Tris/HCl, pH 8.8, 6 M urea, 30% (v/v) glycerol, 2% (w/v) SDS, and 2% (w/v) iodoacetamide] under constant stirring. For the second dimension, the strips were mounted on 10% SDS-PAGE gels and run at 95 V for 2 h in a Mini-Protean 3 system (Bio-Rad). The gels were either stained by Coomassie Blue G-250 or transferred to nitrocellulose membranes.

### 2.6. Production of Polyclonal Anti-gp60 Antibodies

To this purpose, the same methodology reported by Ruiz-Baca et al. [19] was followed. Briefly, several samples of the gp60 antigen from 2D-PAGE gels stained with Coomassie Blue were obtained. The gel pieces were macerated with the help of a mortar and resuspended in sterile distilled water. A dose was intramuscularly injected every 7 days for 4 weeks into male New Zealand rabbits. Each dose contained approximately 12.5 *μ*g of gp60 suspended in a volume of 1 mL (50% distilled water and 50% adjuvant). The first and three last doses contained complete and incomplete Freund's adjuvant (Sigma), respectively. One week after the last dose, rabbits were sacrificed and bled to death, the serum was collected, and immunoglobulins were fractionated with 50% ammonium sulfate.

### 2.7. Western Blot

Immunodetection was carried out as previously described by Ruiz-Baca et al. [20]. Briefly, the membrane was blocked for 1 h with a skim milk solution (5%, w/v) in phosphate-buffered saline (PBS), pH 7.2. After washing thrice with PBS, membrane was incubated overnight with either anti-gp60 rabbit polyclonal antibody diluted 1 : 2000 or sera from nonimmunized or preimmunized mice with gp60 diluted 1 : 100 in PBS supplemented with 0.05% (v/v) Tween 20, as primary antibodies. Membrane was then washed thrice with the same buffer and incubated in anti-rabbit IgG or anti-mouse IgG, both goat horseradish peroxidase-conjugated secondary antibodies, diluted 1 : 1000 in PBS with gentle shaking for 2 h. After washing with PBS, enzyme activity was revealed with a solution containing 3-3′-diaminobenzidine (DAB, 1 mg/mL) and 0.01% (v/v) H_2_O_2_. As a positive control for the presence of anti-gp60 antibodies in mice, anti-gp60 polyclonal antibodies were used.

### 2.8. Immunofluorescence to gp60 in Yeast Cells

Yeast cells were obtained from a 10-day-old culture, washed thrice with PBS, and centrifuged for 5 min at 7000 g. The resultant cell pellet was fixed for 30 min in 2% (w/v) paraformaldehyde diluted in PBS at 4°C and washed four times with cold PBS. Fixed cells were incubated for 1 h at room temperature (RT) with the anti-gp60 polyclonal antibody diluted 1 : 100 in PBS solution containing 5% (w/v) bovine serum albumin (BSA). After washing thrice with PBS, cells were incubated for 1 h at RT in the dark with a goat anti-rabbit antibody conjugated to fluorescein isothiocyanate (FITC), diluted 1 : 50 in PBS solution containing 0.1% (w/v) BSA. Finally, yeast cells were washed thrice with PBS and analyzed using the Axio-observer Z1 LSM 700 Carl Zeiss confocal microscope. Yeast cells processed as described above but incubated either with preimmune serum or with the secondary antibody conjugated to FITC only were used as control. Other cells fixed only in paraformaldehyde were used to assess the potential autofluorescence.

### 2.9. Purification of gp60

For the purification of gp60, CW proteins were extracted as described above. Approximately 6 mg of proteins was resuspended in 18 mL of hydration buffer at a concentration of 7% (v/v) ampholytes with a pH gradient of 3–10. The suspension of proteins was loaded onto a Rotofor preparative IEF cell (Bio-Rad) and ran for 4 h at a constant power of 12 watts (W) using a Powerpac Universal Power Supply (Bio-Rad). Subsequently, fractions enriched in gp60 were pooled and mixed with hydration buffer to reach 18 mL without further addition of ampholytes. This sample was run on the same equipment for 2.5 h at a constant power of 12 W. Fractions enriched in gp60 were precipitated with 70% (v/v) ethanol at −20°C for 24 h and centrifuged and the resulting pellets were resuspended in 2x buffer [0.125 M Tris-HCl, pH 6.8; 4% (w/v) SDS, 20% (v/v) glycerol, 200 mM *β*-mercaptoethanol, and 0.002% (w/v) bromophenol blue]. The samples were separated by continuous elution electrophoresis for 6 h at 1 W in a Mini-Prep Cell (Bio-Rad). Fractions enriched in gp60 were pooled and kept in elution buffer [0.3% (w/v) Tris-HCl, 1.4% (w/v) glycine, and 1% (w/v) SDS]. The glycoprotein was monitored along the steps of purification by SDS-PAGE electrophoresis and Western blot using anti-gp60 as primary antibody.

### 2.10. gp60 Peptide Identification by LC-MS/MS Analysis

The gp60 was purified and manually excised from Coomassie Blue-stained electrophoresis gels. The gel pieces were washed and reduced with DTT and alkylated with iodoacetamide and in gel-digested with trypsin. LC-MS/MS analysis was performed on an Orbitrap Fusion Tribrid (Thermo Scientific) utilizing a nanospray ionization source. An instrument method was used to collect full mass spectrum every three seconds and continuously fragment the most intense ions with 38% collision-induced dissociation (CID) and record the resulting MS/MS spectra. Raw tandem mass spectra were converted to mzXML files and then to Peak List files (PKL)* via *the Transproteomic Pipeline (Seattle Proteome Center, Seattle, WA). PKL were searched using Mascot (Matrix Scientific, Boston, MA) against separate target and decoy databases containing dimorphic fungi proteins (*Sporothrix*,* Coccidioides*,* Blastomyces*,* Paracoccidioides*,* Lacazia*, and* Penicillium marneffei*) downloaded June 17, 2014, from the Dimorphic Fungal Database from Broad Institute and National Center for Biotechnology Information (NCBI). Mascot settings were as follows: tryptic enzymatic cleavages allowing for up to 2 missed cleavages, peptide tolerance of 800 ppm, fragment ion tolerance of 0.8 Da, fixed modification due to carboxyamidomethylation of cysteine (+57 Da), and variable modifications of oxidation of methionine (+16 Da) and deamidation of asparagine or glutamine (+0.98 Da). Statistically significant protein was determined at a 1% protein false discovery rate applied via ProteoIQ (NuSep, Bogart, GA) by loading Mascot.DAT target and decoy search files into the software program.

### 2.11. Effect of gp60 on the Mice Immune Response during* S. schenckii* Infection

To evaluate the effect of gp60, BALB/c and C57BL/6 mice strains were injected intramuscularly with 100 *μ*L (50% elution buffer and 50% adjuvant) containing 2 *μ*g of gp60 every 7 days for three weeks (gp60-preimmunized mice groups); the first and subsequent doses were administered in complete and incomplete Freund adjuvant, respectively. Animals of each strain received the same treatment protocol without adding gp60 (nonimmunized mice groups). One week after treatment completion, blood samples from each mouse were collected in 500 *μ*L tubes by facial vein phlebotomy to determine the anti-gp60 antibodies using the anti-gp60 polyclonal antibodies as a positive control. Thereafter, all mice were infected subcutaneously by injecting 100 *μ*L of PBS containing 5 × 10^5^ conidia of* S. schenckii*. Mice were examined every three days during three weeks by evaluating the thickness of dorsoplantar inflammation with a Mitutoyo® micrometer and the presence of skin ulceration or scarring in foot dorsum. Additionally, five animals of each strain neither infected nor treated with gp60 were used as control groups. 19 days after infection, the mice were bled to determine anti-gp60 antibodies and some cytokines of Th1 and Th2 responses.

### 2.12. Determination of Cytokines

The cytokines IL-1*β*, TNF-*α*, IL-12p40, IL-12p70, MIP-2, and IFN-*γ* from the Th1 response and IL-6, IL-4, and IL-10 from Th2 response were determined using the Luminex xMAP Milliplex Analyst Platform technology (Millipore kit) according to the manufacturer's instructions. Briefly, the serum samples were diluted in the assay buffer contained in the kit at 1 : 1 (v/v) ratio. The quality controls and the cytokine standards provided by the kit were also prepared. Afterwards, a 96-well ELISA plate was washed with washing buffer and 25 *μ*L of each standard, control, and sample was added to the respective wells followed by 25 *μ*L of assay buffer and 25 *μ*L of magnetic beads. The plate was stirred at RT and after 2 h, it was washed twice with washing buffer and 25 *μ*L of detection antibody was added to each well. Following this, the plate was stirred at RT and after 1 h, 25 *μ*L of streptavidin and phycoerythrin was added and incubated for 30 min at RT. Finally, after two additional washes the plate was read on a Magpix reader after adding 150 *μ*L of driving fluid to each well.

### 2.13. Statistical Analysis

Data from cytokine levels are expressed as mean ± SD and analyzed by ANOVA followed by Tukey test. Data from ulceration are expressed as percentages and analyzed by Fisher's test. GraphPad Prism 6 was used to perform all analysis.

## 3. Results

### 3.1. Production of Polyclonal Antibodies

**Figure 1:**
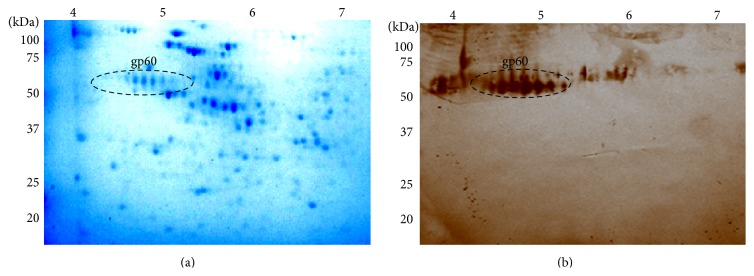
Cell wall proteins of yeast-like cells of* S. schenckii* were separated on 2D-PAGE gels and detected with Coomassie Blue (a) and Western blot using polyclonal anti-gp60 primary antibodies and peroxidase-conjugated secondary antibodies (b). Signal was revealed with a DAB-peroxidase substrate solution. Dotted circles represent gp60 antigen (pH 4.5–5.5) showing several isoforms. The position of molecular weight standards is indicated on the left.

Anti-gp60 polyclonal antibodies were produced to monitor the reactivity of the yeast cell to this antigen and to track gp60 during the purification process.  Figure 1 shows the separation of the CW proteins in 2D-PAGE gels, where at least six isoforms of the glycoprotein are seen in a pH range between 4.5 and 5.5 (Figure 1(a)). These isoforms were cut from different 2D-PAGE gels and used to inoculate a rabbit intramuscularly. One week after the last immunization dose, the rabbit was bled to obtain hyperimmune serum which was analyzed by Western blot (Figure 1(b)). Antibodies titers were up to 1/10000 (data not shown).

### 3.2. Confocal Microscopy

**Figure 2:**
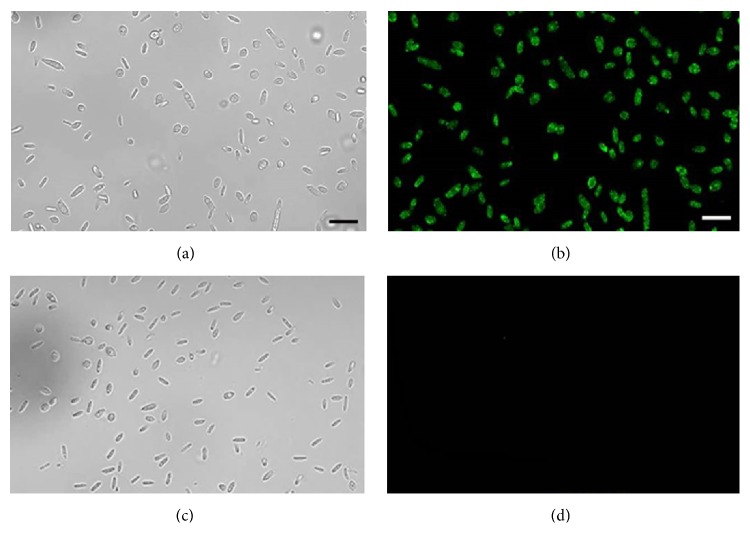
Immunolocalization of gp60 by confocal microscopy in the yeast morphotype of* S. schenckii*. Cells were incubated with polyclonal anti-gp60 antibodies ((a) and (b)) or preimmune sera ((c) and (d)). Bars = 10 *μ*m.

Immunofluorescence and confocal microscopy confirmed the expression of gp60 in the yeast morphotype of the fungus. This morphotype was observed with phase contrast microscopy (Figures 2(a) and 2(c)). Images indicate that gp60 is located on the cell surface of yeast cells (Figure 2(b)). Controls incubated with preimmune sera and paraformaldehyde-associated autofluorescence were negative (Figure 2(d)).

### 3.3. Purification of gp60

**Figure 3:**
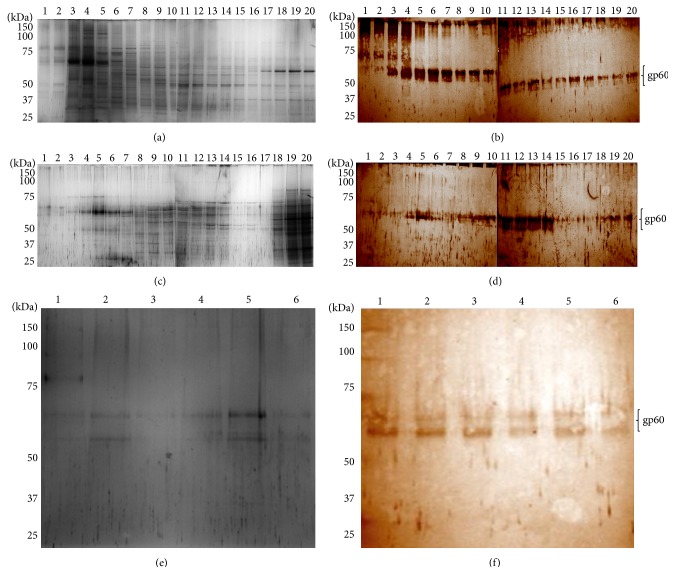
Purification of gp60. Cell wall proteins of yeast-like cells of* S. schenckii* were separated by sequential steps of liquid phase isoelectric focusing and electrophoresis with continuous electroelution as described in Materials and Methods. After each step, fractions were analyzed by SDS-PAGE ((a), (c), and (e)) and Western blot ((b), (d), and (f)) using polyclonal anti-gp60 primary antibodies and peroxidase-conjugated secondary antibodies. Signal was revealed with a DAB-peroxidase substrate solution. Fractions separated after the first ((a) and (b)) and second ((c) and (d)) liquid phase isoelectric focusing steps and after electrophoresis with continuous electroelution ((e) and (f)).

In order to obtain sufficient amounts of purified gp60 to determine its effect on the immune response, we standardized a purification protocol based on liquid phase IEF and continuous elution electrophoresis. The protein was monitored through the various purification steps by Western blot assays using the anti-gp60 antibodies. Results from liquid phase IEF showed that most of the pollutant proteins were in fractions 3 and 4, and despite the fact that gp60 was detected in every fraction, results showed a higher antigen concentration in fractions corresponding to lanes 5 through 11 (Figures 3(a) and 3(b)). These fractions were pooled and again subjected to IEF, which showed a higher concentration of gp60 in fractions corresponding to lanes 11 through 14 (Figures 3(c) and 3(d)). Subsequently, enriched fractions obtained after the second IEF were pooled and the pool was purified by continuous elution electrophoresis. This step yielded 5 fractions containing gp60 with a high degree of purity (Figures 3(e) and 3(f), lanes 2, 3, 4, 5, and 6) with the different isoforms ranging from 55 to 65 kDa.

### 3.4. Peptide Sequencing of gp60

The spots of the different gp60 isoforms separated in 2D-PAGE gels stained with Coomassie Blue were cut from the gel and exposed to tryptic digestion. The resulting peptides were analyzed by mass spectrometry (LC-MS/MS) and gp60 was identified as a carboxy-*cis*,* cis*-muconate cyclase of* S. schenckii* (Table 1).

### 3.5. Effect of gp60 on the Mice Immune Response during* S. schenckii* Infection

**Figure 4:**
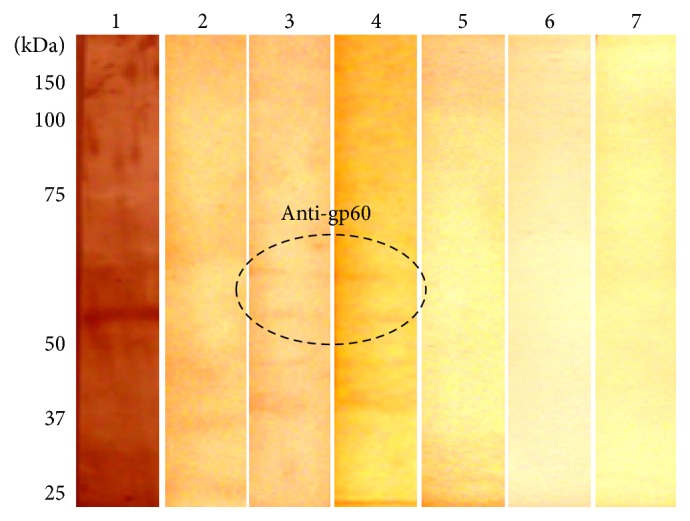
Presence of anti-gp60 antibodies in mice prior to infection. Cell wall proteins of yeast-like cells of* S. schenckii* were analyzed by Western blot using sera from nonimmunized and gp60-preimmunized mice and peroxidase-conjugated secondary antibodies. Signal was revealed with a DAB-peroxidase substrate solution. Positive control (lane 1), nonimmunized C57BL/6 (lane 2), gp60-preimmunized C57BL/6 (lanes 3 and 4), nonimmunized BALB/c (lane 5), and gp60-preimmunized BALB/c (lanes 6 and 7) mice.

**Figure 5:**
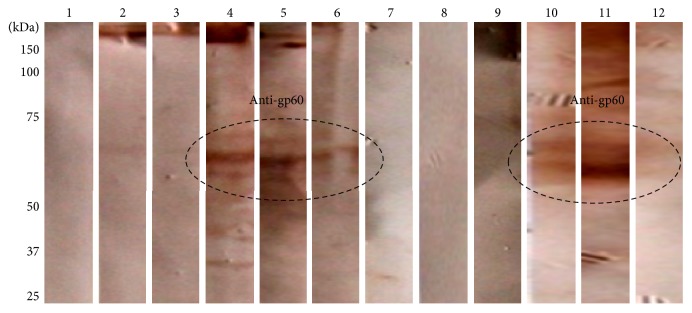
Presence of anti-gp60 antibodies in nonimmunized and gp60-preimmunized mice after 19 days after infection. Cell wall proteins of yeast-like cells of* S. schenckii* were analyzed by Western blot using sera from nonimmunized and gp60-preimmunized mice and peroxidase-conjugated secondary antibodies. Signal was revealed with a DAB-peroxidase substrate solution. Nonimmunized C57BL/6 (lanes 1–3), gp60-preimmunized C57BL/6 (lanes 4–6), nonimmunized BALB/c (lanes 7–9), and gp60-preimmunized BALB/c (lanes 10–12) mice.

The gp60-preimmunized C57BL/6 and BALB/c groups were subsequently infected with* S. schenckii*. Only gp60-preimmunized C57BL/6 mice showed the presence of anti-gp60 antibodies prior to infection, although detection was very low as can be seen in Figure 4 (lanes 3 and 4). Nonimmunized groups of both strains did not produce anti-gp60 antibodies (Figure 4, lanes 2 and 5). However, 21 days after infection, gp60-preimmunized mice of both strains, as well as those that were infected only (nonimmunized mice groups), showed anti-gp60 antibodies. A higher detection was shown by the groups that received gp60 before infection (Figure 5, lanes 4, 5, 6, 10, 11, and 12) compared to the nonimmunized groups (Figure 5, lanes 1, 2, 3, 7, 8, and 9), particularly the C57BL/6 strain (Figure 5, lanes 4, 5, and 6).

**Figure 6:**
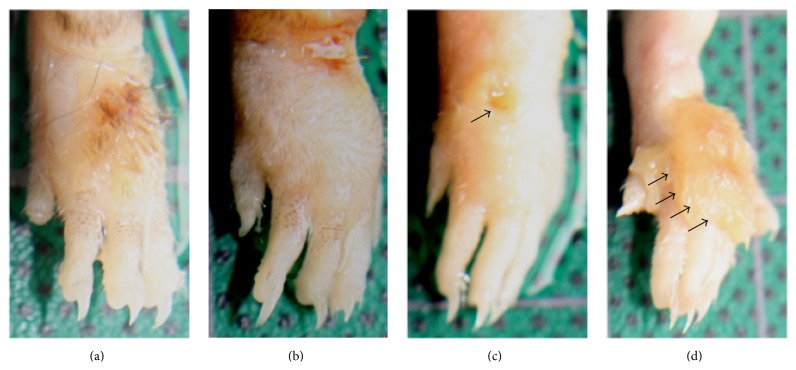
Ulceration at the foot dorsum in nonimmunized and gp60-preimmunized mice. Nonimmunized C57BL/6 (a), gp60-preimmunized C57BL/6 (b), nonimmunized BALB/c (c), and gp60-preimmunized BALB/c (d). Arrows indicate ulceration sites.

**Figure 7:**
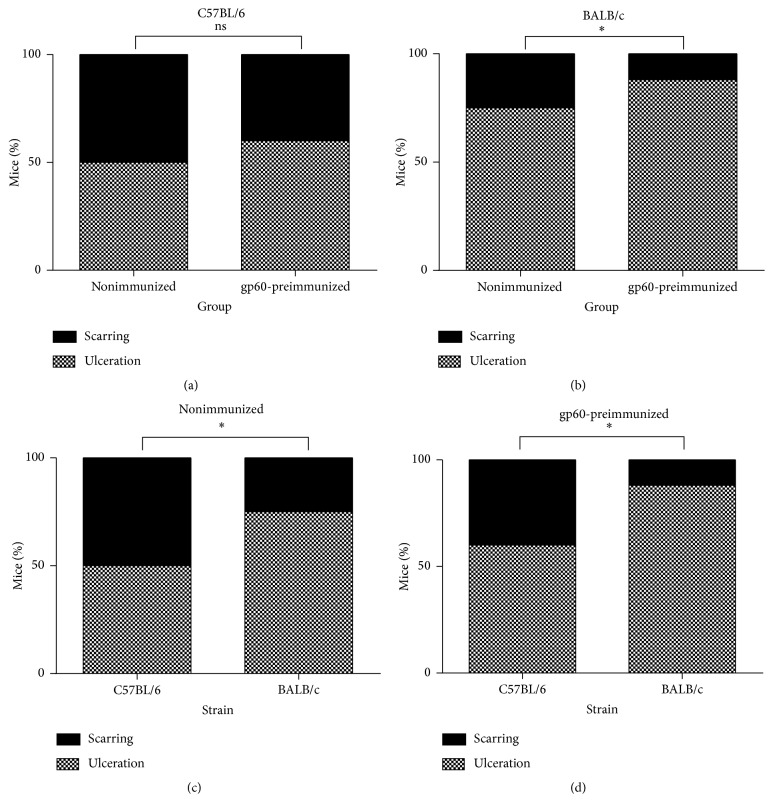
Statistical analysis of foot dorsum ulceration in mice. Nonimmunized and gp60-preimmunized C57BL/6 groups (a), nonimmunized and gp60-preimmunized BALB/c groups (b), nonimmunized groups (c), and gp60-preimmunized groups (d). Data are presented as percentages. ns: no significant differences;  ^*∗*^significant differences between groups (*P* < 0.05).

Nonimmunized mice of both strains seemed to heal faster than those preimmunized with gp60 prior to infection (Figure 6); however, nonimmunized C57BL/6 mice (Figure 6(a)) did not show significant differences in foot dorsum ulceration (Figure 7(a)) as compared to those of the same strain that received gp60 prior to infection (Figure 6(b)), contrary to nonimmunized BALB/c mice (Figure 6(c)) which showed significant differences (Figure 7(b)) compared to gp60-preimmunized mice of the same strain (Figure 6(d)). A lower susceptibility to* S. schenckii* was noted in C57BL/6 mice, since nonimmunized C57BL/6 mice showed lower ulceration than nonimmunized BALB/c mice (Figure 7(c)). Also, a different effect induced by gp60 was noted between the strains since gp60-preimmunized C57BL/6 mice showed a significant lower ulceration than gp60-preimmunized BALB/c mice (Figure 7(d)). No statistical differences in inflammation were detected between nonimmunized and gp60-preimmunized groups (data not shown).

### 3.6. Cytokine Profiles during Infection by* S. schenckii*

**Figure 8:**
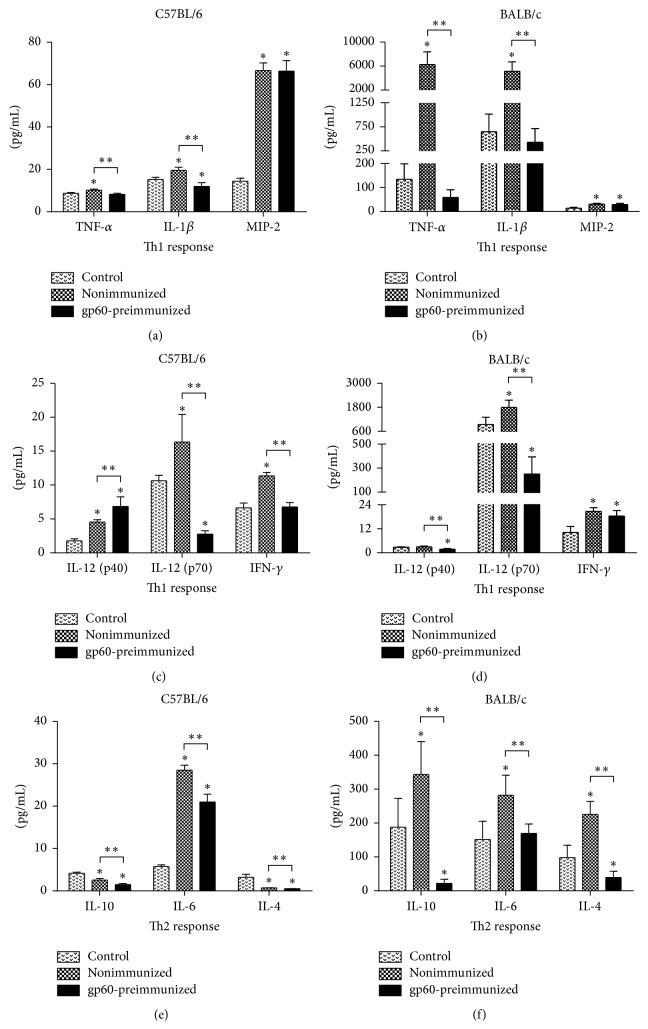
Cytokine profiles of control, nonimmunized, and gp60-preimmunized groups. C57BL/6 Th1 ((a) and (c)), BALB/c Th1 ((b) and (d)), C57BL/6 Th2 (e), and BALB/c Th2 (f). Data show mean values ± SD (*n* = 10 for each group and *n* = 5 for controls).  ^*∗*^Significant differences compared to control (*P* < 0.05).  ^*∗∗*^Significant differences between groups (*P* < 0.05).

The cytokine profiles expressed by C57BL/6 and BALB/c mice strains were different in the baseline levels and during infection with* S. schenckii.* Control C57BL/6 mice showed lower levels of cytokines such as TNF-*α*, IL-1*β*, and IL-12 (p70) compared with control group of BALB/c (Figure 8), which suggests a predisposition towards the inflammatory or Th1 response in BALB/c mice.

**Figure 9:**
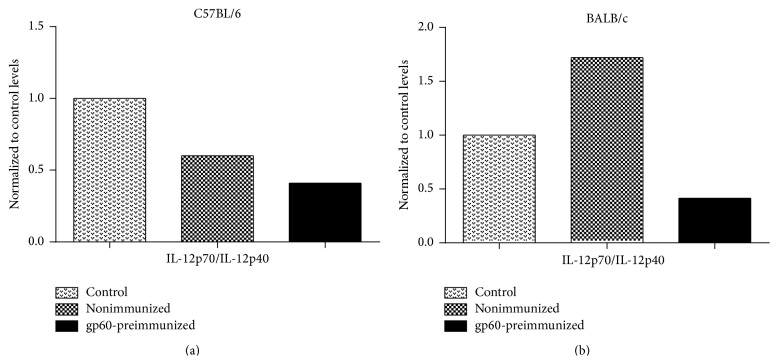
IL12-p70/IL-12p40 ratios. Values of C57BL/6 (a) and BALB/c (b) groups were normalized to corresponding control levels.

The nonimmunized C57BL/6 group showed significantly higher levels of TNF-*α* and IL-1*β* as compared to the control group. Likewise, the level of MIP-2 was 5-fold higher in nonimmunized mice compared to the control group (Figure 8(a)). In the case of nonimmunized BALB/c mice, the levels of TNF-*α* and IL-1*β* increased 45- and 8-fold over the control value, respectively (Figure 8(b)), that is, much more than in nonimmunized C57BL/6 group. On the contrary, the levels of MIP-2 increased about 2-fold over the baseline levels (Figure 8(b)), which is about half the increase observed in nonimmunized C57BL/6 mice. In nonimmunized C57BL/6 mice, the IL-12 (p70)/IL-12 (p40) ratio decreased as compared to the control group (Figure 9(a)), contrary to the levels of IFN-*γ*, which increased significantly (Figure 8(c)). In contrast to C57BL/6, nonimmunized BALB/c mice showed an increase of almost twice in the IL-12 (p70)/IL-12 (p40) ratio as compared to the control group (Figure 9(b)), and the same occurred with IFN-*γ* (Figure 8(d)). Regarding the cytokines of the Th2 response, nonimmunized C57BL/6 mice showed a significant decrease in the levels of IL-10 and IL-4 with respect to the control group, but the levels of IL-6 increased almost 5-fold (Figure 8(e)). In nonimmunized BALB/c group, the levels of IL-10 and IL-6 increased almost twice, whereas the level of IL-4 increased more than twice over the control group (Figure 8(f)); however, although the levels of the Th2 response increased in nonimmunized BALB/c mice, the increase was not as high as that of the cytokines characteristic of Th1.

### 3.7. Effect of gp60 on Cytokine Profiles during Infection with* S. schenckii*

In the gp60-preimmunized C57BL/6 group, the levels of TNF-*α* and IL-1*β* decreased significantly when compared to the nonimmunized group; accordingly, the amount of TNF-*α* reached baseline levels in the control group, while that of IL-1*β* was below the control value (Figure 8(a)). On the other hand, MIP-2 remained in the same level as the nonimmunized group; significant differences were observed only after comparing with the control group (Figure 8(a)). Cytokine levels in gp60-preimmunized BALB/c mice decreased more markedly. The levels of TNF-*α* dropped more than 100-fold with respect to the nonimmunized group, falling even below the control levels, yet no significant differences were observed when compared with the control group. The level of IL-1*β* decreased almost 12-fold as compared to nonimmunized mice whereas the level of MIP-2 was unaffected as compared to the nonimmunized group, remaining above the level of the control group (Figure 8(b)). It is noteworthy that in both strains the levels of MIP-2 did not change in any of the gp60-preimmunized groups compared to the nonimmunized groups.

In gp60-preimmunized C57BL/6 mice, the IL-12 (p70)/IL-12 (p40) ratio decreased compared to the nonimmunized and control groups (Figure 9(a)). IFN-*γ* also decreased significantly (to control levels) compared to nonimmunized group (Figure 8(c)). In gp60-preimmunized BALB/c mice, the IL-12 (p70)/IL-12 (p40) ratio decreased 4-fold and 2-fold compared to the nonimmunized and control groups, respectively (Figure 9(b)), and the level of IFN-*γ* also decreased, though not significantly compared to nonimmunized group (Figure 8(d)). Although a slight decrease in IFN-*γ* was observed in gp60-preimmunized mice, gp60 appears to have a greater effect on the IL-12 (p70)/IL-12 (p40) ratio, decreasing the heterodimeric form and increasing the homodimeric one. Furthermore, the levels of IL-10 decreased significantly in gp60-preimmunized C57BL/6 mice compared to nonimmunized group, falling even below that control level. The level of IL-6 also decreased, yet it remained above the control level. The level of IL-4 also decreased significantly compared to nonimmunized and control groups (Figure 8(e)). It is worth noting that although the levels of both Th1 and Th2 cytokines decreased in gp60-preimmunized C57BL/6 mice, they were not very affected in terms of ulceration (Figures 6(b) and 7(a)) being as damaged as nonimmunized C57BL/6 mice (Figure 6(a)). In gp60-preimmunized BALB/c mice, the level of IL-10 decreased over 17-fold as compared to nonimmunized group, reaching levels below baseline values. The levels of IL-6 and IL-4 also decreased to the control level (IL-6) and below the control level (IL-4) (Figure 8(f)).

## 4. Discussion

In the present work, gp60 from CW of* S. schenckii *was purified by isoelectric focusing and continuous elution electrophoresis, and its effects over the immune response in an experimental subacute sporotrichosis model in two mice strains were evaluated.

To isolate this glycoprotein as a single antigenic peptide, a purification protocol which takes advantage of the IP and molecular mass was used. The purified gp60 was sequenced by tandem mass spectrometry, yielding a sequence homologous to secreted gp70 [22], a carboxy-*cis*,* cis*-muconate cyclase involved in the *β*-ketoadipate pathway required for the catabolism of aromatic compounds [23]. However, unlike gp60, which was sequenced from the form present in the cell surface, the sequence of gp70 was obtained from the secreted, extracellular protein, which might confer differences in the immune mechanisms induced by each antigen, as it has been demonstrated with the BAD-1 protein of* Blastomyces dermatitidis*; accordingly, the CW form induces regulatory mechanisms of the immune response through the production of TGF-*β*, whereas the secreted counterpart independently regulates from this cytokine [24].

The identification of gp60 as an enzyme complicates its implication in the immunological mechanisms. However, several CW enzymes such as the *β*-1,3-glucosyltransferase of* Coccidioides posadasii* [25] and glyceraldehyde-3-phosphate dehydrogenase (GAPDH) from* Paracoccidioides brasiliensis* [26], altogether with other enzymes of pathways such as glycolysis and the Krebs and glyoxylate cycles, are classified as moonlight or multifunctional proteins due to their participation in pathogenic processes independently of their metabolic activities [27].

Confocal microscopy, using a rabbit polyclonal antibody against gp60 (Figure 1(b)), demonstrated that this protein is distributed along the cell surface of the yeast morphotype (Figure 2(b)). Location of gp60 implicates that it could be involved in different potential roles as protective, immunosuppressive, immunostimulatory, or adhesive proteins. The 60 kDa heat shock protein (Hsp60) of* Histoplasma capsulatum* has been associated with protection against stress conditions, since it is expressed when the yeast is subjected to thermal stress, acting as chaperone of proteins with essential functions [28]. The 120 kDa protein present on the surface of* B. dermatitidis* has been proposed as a modulator of the immune response, since it is capable of inhibiting the inflammatory response through the production of TGF-*β* [29], contrary to the glycoprotein of the outer wall of the spherules of* C. immitis* and* C. posadasii*, which acts as an immunodominant antigen capable of inducing cellular and humoral responses during parasitic infections [30, 31].* P. brasiliensis* GAPDH and a 30 kDa protein act as adhesins and have an important role in the interaction between the fungus and the host cells [26, 32]. In the same line, gp70 of* S. schenckii* has been reported to have a role as an adhesin associated with the virulence of* S. schenckii* strains [16, 33]. However, an increased expression of this glycoprotein has been associated with less virulent strains of* S. brasiliensis* [34]. Therefore, the presence of gp60 on the cell surface and the homology with gp70 suggest a role in immune and adhesion mechanisms.

In the present work, both mice strains expressed different cytokine profiles. Their analysis in nonimmunized, gp60-preimmunized, and control mice groups demonstrated that levels of Th1 and Th2 response cytokines in C57BL/6 mice remained low compared to those of BALB/c mice, except for MIP-2 which was slightly higher in C57BL/6 mice. The importance of MIP-2 lies in its ability to attract neutrophils to the infected areas in the early stages of the immune response, regardless of the type of pathogen [35]. Moreover, it is known that the levels of MIP-2 change as a proportional function of IL-6, with a direct relationship between them [36]. The concentration of IL-6 noticeably increased in nonimmunized C57BL/6 mice compared to the controls. Besides being associated with the Th1 response, IL-6 promotes the growth and differentiation of B lymphocytes stimulating the humoral response, which suggests a slight tendency of the C57BL/6 mice to the Th2 response, which probably explains the stronger antibody response in this strain. IL-6 is also involved in the activation of the Th17 response, characterized by neutrophil recruitment and inflammation [37]; thus, it is not entirely clear what type of immune response in C57BL/6 mice is relevant to confront infection by* S. schenckii*. On the other hand, nonimmunized BALB/c mice produced higher amounts of proinflammatory cytokines or Th1 response, as indicated by the levels of TNF-*α*, IL-1*β*, and IL-12p70. These mice exhibited severe tissue damage at the foot dorsum. In fact, increased levels of TNF-*α* have been associated with the severity of sporotrichosis skin lesions in BALB/c mice [38]. Moreover, more severe skin lesions in BALB/c than in C57BL/6 mice correlate with higher IL-4 levels observed in BALB/c mice, according to models of* P. brasiliensis* infection in which elevated levels of IL-4 are related to a worsening of the infection. This is probably due to a decreased fungicide capacity of lung phagocytes from normal mice compared with that of phagocytes from IL-4-deficient mice [39].

The low levels of IL-10 observed in nonimmunized C57BL/6 mice compared to nonimmunized BALB/c may explain why lower tissue damage was noted in C57BL/6 mice, as has been reported in infections caused by* P. brasiliensis* where IL-10 deficiency leads to increased immunity and regressive infection without enhancing tissue pathology [40]. In this sense, it is worth noting that the amount of specific antibodies against gp60 was higher in the C57BL/6 strain than in the BALB/c strain. The gp60 isoforms ranged between 60 and 70 kDa. Accordingly, sera from patients with sporotrichosis contain antibodies recognizing* S. schenckii *antigens in a range of 40–70 kDa [41]. Furthermore, a seroprevalence of the 60–70 kDa antigen exists, and patients with this mycosis contain serum antibodies against such a protein [22]. This immunodominant 60–70 kDa antigen also has been demonstrated in feline sporotrichosis, suggesting its potential as a marker for diagnosis or as a candidate for the development of therapeutic vaccines [42]. Humoral response in mice infected with species from the* S. schenckii* complex shows an antibody production pattern similar to human and cat patterns, where 60–70 kDa recognition becomes a constant [21]. This suggests a convergent humoral response between the three mammals species hosts that may increase our understanding of the coevolution of these hosts with* S. schenckii *species [42]. Whereas the Th2 response is associated with fungal susceptibility, it is known that antibodies can affect the balance of cytokines and the induction of regulatory T cells that help to reduce tissue damage caused by exacerbated inflammatory responses [43].

The conidial morphotype was used in our model of* S. schenckii* infection. This fact should be considered in the interpretation of mice immune response, along with the route of infection, the concentration and virulence of the inoculum, the mouse strain used, and its genetic background.* S. schenckii* yeast and conidial morphotypes are recognized by different receptors [44]. The mannose receptor is the one that recognizes the conidial morphotype, so a different expression of this receptor in each mouse strain may determine a difference in immune response, even though this receptor can activate both inflammatory and regulatory pathways [37]. Furthermore, the magnitude of the response may also vary according to the morphotype, as yeasts coincubated with mast cells have been unable to induce degranulation in* in vitro* assays, contrary to what occurs in conidia, which supports the increased immunological activity of the conidial morphotype [45].

Results of the present study emphasize the lack of a detailed knowledge of the mechanisms of pathogenicity of the different species leading to the development of sporotrichosis. Preimmunization of C57BL/6 and BALB/c strains with gp60 resulted in a downregulation effect on both strains of mice whose cytokine levels were lower as compared to the nonimmunized, particularly in the case of BALB/c. These results could explain why monoclonal antibodies directed against other* S. schenckii* antigens such as gp70 have an immunoprotective effect in murine models [46]. Noteworthy, antibodies against gp70 are not the only ones which have a protective role against* S. schenckii* infection, since passive transference of sera from mice containing antibodies against a 44 kDa hydrolase and a 47 kDa enolase has shown a protective role during murine sporotrichosis [47].

It is worth noting that gp60-preimmunized C57BL/6 mice seemed to be able to resolve the infection as nonimmunized mice. It has been demonstrated that dendritic cells from C57BL/6 mice incubated with* S. schenckii* promote combinations of Th1 and Th17 responses [48]. A recent* in vitro* assay suggests that Th1/Th17 combined responses against* S. schenckii* depend on IL-23 [49]. Notably, IL-17 is involved in neutrophil recruitment and in the production of MIP-2; a cytokine markedly increased in nonimmunized and gp60-preimmunized C57BL/6 mice. Thus, gp60 does not seem to have an effect on the Th17 response. Indirectly, we may infer that C57BL/6 mice tend to assemble Th17 response after infection with* S. schenckii*. Dendritic cells increase IL-23 production after ligation of *β*-glucans by the dectin-1 receptor regulating fungal pathogenicity via Th-17 responses [50]. Furthermore, vaccination with attenuated yeast cells of primary pathogenic fungi such as* B. dermatitidis*,* C. posadasii*, and* H. capsulatum* induces protective Th17 responses against a lethal infection. Protection is impaired when IL-17 levels are decreased with specific monoclonal antibodies [51]. Galectin-3 receptor-deficient mice express higher levels of Th17 response cytokines as TGF-*β*1, IL-23, IL-17, and IL-6, in a model of* H. capsulatum* infection [52]. Thus, it could be hypothesized that C57BL/6 immunity tendency towards Th17 response, as it was observed in our study, might be related to recognition of* S. schenckii* conidia through the differential expression between C57BL/6 and BALB/c mice of the galectin-3 receptor and its ligand.

BALB/c strain cytokine levels were greatly affected by gp60. Previous reports indicate that BALB/c mice infected with* S. schenckii* express a Th1 response mediated by ligation of TLR-2 and TLR-4 receptors [9, 10, 53, 54]. Probably, preimmunization with gp60 blocks one of these receptors and prevents activation signaling pathways that involve NF-kB transcription factor. Additionally, gp60 may have interfered with caspase-1-dependent signaling pathways activation, which has proven to be important for the production of Th1 cytokines during experimental sporotrichosis [55, 56]. Resistance to paracoccidioidomycosis in mice is related to paracoccin TLRs activation that triggers a balanced Th1 immunity [57]. Furthermore, during* S. schenckii *antigens recognition, the lack of TLR-2 and TLR-4 receptors, combined with the presence of these surface antigens, stimulates TGF-*β*-mediated regulatory responses with inhibition of Th1 response [53, 54]. Decreased levels of Th1 cytokines observed in gp60-preimmunized mice could be related to these mechanisms whose balance determines the efficiency of Th1 immune response mediated by cytokines.

The IL-12-p70/IL-12-p40 ratio decreased in gp60-preimmunized mice, mainly in the BALB/c strain, indicating the presence of a downregulation effect compared with nonimmunized mice since IL-12 p70 is considered the bioactive one. This cytokine is produced only after dendritic cells are primed with microbial stimuli that upregulate IL-12 p40, which stimulates CD40 expression. CD40 ligation induces IL-12 p35, which finally yields bioactive IL-12 p70 [58]. The relevance of IL-12 as a protective cytokine has been demonstrated in gerbils infected with* S. schenckii* [59] but also as linker between innate and adaptive responses mediated by dendritic cells after* S. schenckii* phagocytosis [60]. Thus, gp60 might block one of the steps that lead the production of the bioactive form of this cytokine, avoiding the development of a full Th1 response.

Finally, it seems that gp60 has a greater downregulation effect on the Th1 response, since C57BL/6, a strain that probably depends on a different response like Th17 to fight this fungus, was not as affected as BALB/c strain, which appears to be dependent on Th1 response in order to clear the infection with* S. schenckii*.

## Figures and Tables

**Table 1:** Analysis of gp60 by LC-MS/MS.

Protein name	Molecular weight (kDa)	Protein score	Peptide sequence	# of spectra	Sequence coverage (%)
Carboxy-*cis*, *cis-*muconate cyclase [*S. schenckii*]	43.3	1171.6	LVEMSLVNAEIIGEPIDLTTFNTDPGLTEIR AGVSCASYSWYGLGPFDELR TVIPGQDATCWVAICPATHTAFVTDIR AVYVTSNTEHNSVVAIPIAR GGNGINPR NGSLLLNHATSTATGGR KPVQHALLTPLGLDR VTVVGEPAELPGEFPTTVGASDKFNLVCVGLTGAK	185	42

Mass spectra were analyzed with Mascot and validated with ProteoIQ software.

Protein name: protein name as deduced by comparing peptide sequences via the software BLAST.

Molecular weight (kDa): theoretical molecular mass predicted from the amino acid sequence of the identified protein.

Protein score: sum of Mascot ion scores of all nonredundant peptides belonging to the protein.

Peptide sequence: peptides identified after LC-MS/MS analysis.

# of spectra: total number of spectra matched to the protein.

Sequence coverage: coverage of the amino acid sequence of the identified protein.
